# Use and implications of the Apgar score in evaluating resuscitation of newborns with birth asphyxia in a lower-middle-income country

**DOI:** 10.7189/jogh.15.04244

**Published:** 2025-09-19

**Authors:** Jayashree Ramasethu, Indira Narayanan, Jeffery Kodjo Arhin, Rita Fosu Yeboah, Genevieve Insaidoo, Eunice Mintah, Evans Awutey

**Affiliations:** 1 Division of Neonatal Perinatal Medicine, MedStar Georgetown University Hospital, Washington DC, USA; 2 Julius Center for Health Sciences and Primary Care, University Medical Center Utrecht, Netherlands; 3 Kumasi South Hospital, Kumasi, Ghana; 4 Holy Family Hospital, Nkawkaw, Ghana; 5 Methodist Faith Healing Hospital, Ashanti region, Ghana; 6 Krachi West Municipal Hospital, Kete-Krachi, Ghana

## Abstract

**Background:**

The Helping Babies Breathe (HBB) programme has been used worldwide to address neonatal mortality due to birth asphyxia in low resource countries. We aimed to use the Apgar score to evaluate the impact of the programme on neonatal mortality in three district and one regional hospital in Ghana, a lower middle-income country.

**Methods:**

We used Apgar scores as an objective measure of newborn infants’ condition soon after birth and their response to resuscitation, with the assessment carried out primarily by midwives who were trained in HBB. We analysed correlations between Apgar scores and mortality in newborns ≥34 weeks gestation who had birth asphyxia (BA), *i.e.* a one-minute Apgar score <7, and severe BA, *i.e.* a one-minute Apgar score ≤3.

**Results:**

Over the 18-month period from October 2019 to March 2021, 12 995 newborns were delivered at ≥34 weeks gestation or with a birth weight of at least 2000 grams. There were 12 702 live births and 293 stillbirths, of which 134 were intrapartum stillbirths. Among the live births, 2387(18.9%) had BA, including 352 (2.8%) who had severe BA. There was no significant difference in the trend of cases of severe BA or deaths due to BA in the four hospitals, either individually or combined, but there was a 55% decline in intrapartum stillbirths, from 1.6% to 0.89% (*P* = 0.03). Although many babies with BA showed improvement in Apgar scores with resuscitation efforts, the mortality rate among 352 newborns with severe BA was 15.6% – twenty times higher than in the 2045 newborns with a one-minute Apgar score of 4–6, among whom 0.78% died (*P* < 0.001). The mortality rate in newborns with severe BA was higher in those whose scores remained ≤3 than in those whose scores rose to 4–6 or more at five minutes (odds ratio = 19.93, 95% confidence interval = 9.4–42.1, *P* < 0.0001).

**Conclusions:**

The Apgar score provides valuable information about where additional interventions may decrease BA related neonatal mortality in low- and middle-income countries.

Birth asphyxia (BA), defined as the failure to establish breathing at birth, is estimated by the World Health Organization (WHO) to account for 900 000 deaths each year globally and is one of the primary causes of early neonatal mortality [1]. The American Academy of Pediatrics (AAP) basic resuscitation programme, known as Helping Babies Breath (HBB), has been used to improve resuscitation at birth in more than 80 low-resource countries, focussing on the simpler steps of neonatal resuscitation that can be readily applied by midwives at various levels. It promotes the concept that the most important step in resuscitating newborns who do not start breathing on their own, despite drying and stimulation, is positive-pressure ventilation, which should be initiated within one minute after birth [2]. A blended learning quality improvement programme was developed between Georgetown University Medical Centre and MedStar Georgetown University Hospital in collaboration with seven district and one regional hospital in Ghana to improve the quality of neonatal care, with BA selected as the major priority for quality improvement (QI) by the staff in four hospitals in Ghana. The WHO Regional Office for South-East Asia’ point of care QI approach was used within the programme to evaluate the impact of basic neonatal resuscitation, provided through HBB, on the mortality of infants with BA in the four hospitals [3].

## METHODS

This observational study is a secondary analysis of an 18-month QI initiative from October 2019 to March 2021 for reducing mortality due to BA in three district and one regional hospital in Ghana. Hospital staff had already received in-country, in-person training in the AAP HBB course before October 2019. While neither the AAP Neonatal Resuscitation Program [4] nor the HBB programmes describe or utilise the Apgar score, hospitals in Ghana record it routinely in facility births [5]. We therefore used the score as an objective assessment of the condition of the newborn soon after birth, the response to resuscitation, and to evaluate the impact of the HBB program on mortality in newborns with BA. Here we defined BA as an Apgar score <7 at one minute and severe BA as an Apgar score ≤3 at one minute.

The blended learning programme included online lectures, interactive discussions, and case studies. A scheduled in-country workshop was cancelled due to the COVID-19 pandemic. Georgetown University faculty continued to work with the doctors, midwives, and nurses in the four hospitals to initiate and support QI activities virtually using a video communication platform (Zoom), teleconferencing, and a cloud storage service for sharing documents (Google Drive). Midwives performed resuscitation, with neonatal unit staff providing additional support when required and feasible.

The four facilities were Kumasi South Hospital, a regional centre for the Ashanti region, Methodist Faith Healing Hospital; a nonprofit referral hospital for two districts within the Ashanti Region; Krachi West District Hospital in the Oti Region; and Holy Family Hospital, Nkawkaw, another non-profit facility in the Eastern Region. We refer to them as hospitals A, B, C, and D here, in no particular order, to maintain confidentiality. The study was considered exempt from ethical review by the Georgetown University Institutional Review Board. We were also informed that QI initiatives are an integral activity at all of the hospitals and therefore do not require IRB approval from the Ghana Health Service. Permission was granted by the four hospital administration departments to share data, masking patient identifiers, with the other participating hospitals and for presentation and publication in the public forum.

The objective of the QI program was to improve the outcome including the response to resuscitation and mortality due to BA by improving the quality of resuscitation, focusing on term and late preterm newborns (≥ 34 weeks gestational age). The specific, measurable, achievable, relevant, and time-bound (SMART) aim was to increase the proportion of newborns ≥34 weeks’ gestation in whom the Apgar scores were <7 at one minute of life to ≥7 at five minutes by 15% over six months. Interventions included setting up a QI team in each hospital, refresher courses on HBB and Apgar scoring, regular practice on a manikin, raising awareness of the importance of having at least two trained persons at each delivery or achieving this in practice where feasible, and verifying appropriate equipment was kept ready at each delivery. These interventions were implemented from January 2020 after baseline data were collected between October and December 2019. Team leads in each hospital performed random audits to ensure that all the interventions were being carried out and that Apgar scoring was appropriate. Results from the QI activities were shared and discussed weekly or biweekly with the participants from the hospitals, together with challenges, lessons learned, and remedial actions, facilitated by the Georgetown University Medstar Hospital neonatal team (JR, IN, and JA). The bundle of activities implemented in cross-functional interactive QI teams served as the core intervention expected to improve resuscitative measures in the participating health facilities.

Baseline data was collected from October to December 2019 (pre-intervention period) and then monthly through March 2021, with verification done by unit heads. De-identified patient data (*i.e.* without identifiers such as name, date of birth, birth weight, sex, or medical record numbers) from each hospital included the total number of live and stillbirths among infants of a gestational age ≥34 weeks or birth weight at least 2000 grams when gestational age was unknown, the number with BA or severe BA, survival outcomes of these newborns, and the monthly changes in the proportion of infants whose Apgar scores rose from <7 at one minute to ≥7 at five minutes. Intrapartum stillbirths were defined as those with no signs of life at birth (no audible heart rate, breathing, movement, or response to stimulus), no response to resuscitation efforts, and no signs of maceration. Newborns who had an Apgar scores of 0 at one minute and higher scores (≥1) in response to resuscitation, even if transient, were considered to be live births.

### Data analysis

We used chi-squared tests, analysis of variance, and paired *t*-test to explore the differences in BA cases and mortality over the duration of the study. We calculated odds ratios (ORs), standard errors, and 95% confidence intervals (CIs) according to Altman [6]. A *P*-value of <0.05 indicated statistical significance. We performed all analyses in SPSS, version 28.0 (IBM Corp., Armonk, New York, USA).

## RESULTS

Over the 18-month period from October 2019 to March 2021, there were 12 889 deliveries of 12 995 newborns ≥34 weeks gestation or a birth weight of at least 2000 grams, including 53 pairs of twins. There were 12 702 live births and 293 stillbirths, of which 134 were intrapartum stillbirths (10.3 per 1000 births) **(****Table 1****).** Among the live births, 2397 (18.9%) had BA (one-minute Apgar score <7) and 352 (2.8%) had severe BA (one-minute Apgar score ≤3). Seventy-one (2.9%) of the the 2397 newborns with BA died, compared to 55 (15.6%) among the 352 with severe BA (*P* < 0.001). There was significant variation in the incidence of BA and severe BA between the four hospitals (*P* < 0.05). Although hospital C had the largest proportion of cases of BA (25.9%), the mortality in newborns with BA and severe BA was highest in hospital D (*P* < 0.001). Hospital (B) had no deaths among the newborns with BA, as all critically ill newborns were transferred to another hospital. There were 71 deaths among the newborns with BA among the 10 155 live births in the other 3 hospitals (6.99 per 1000 live births).

**Table 1:** Live and stillbirths, incidence of BA, and mortality among newborns ≥34 weeks gestation*

Hospital	Live births	Stillbirths	Intra-partum stillbirths	BA (% of BA cases/live births)	One-minute Apgar score ≤3 (% of severe cases/live births)	One-minute Apgar score 4–6 (% of cases/live births)	Deaths among newborns with BA (%)	Deaths among newborns with one-minute Apgar score ≤3 (%)
A	4039	92	54	757 (18.7)	113 (2.8)	644 (15.9)	21 (2.8)	17 (15)
B	2547	75	27	378 (14.8)	43 (1.7)	335 (12.9)	0	0
C	4010	71	30	1037 (25.9)	113 (2.8)	924 (23.0)	27 (2.6)	16 (14.1)
D	2106	55	23	225 (10.7)	83 (3.9)	142 (6.7)	23 (10.2)	22 (26.5)
Total	12702	293	134	2397 (18.9)	352 (2.8)	2045 (16.1)	71 (2.9)†	55 (15.6)†

BA – birth asphyxia

*BA is defined as a one-minute Apgar score <7.

†*P <* 0.001.

The incidence of BA fluctuated from quarter to quarter in the four hospitals. Hospital A had a significant decrease in the incidence of BA from 25% in the pre-intervention period to 15% by March 2021 (*P* = 0.025); there was no significant change in the incidence of BA in the other hospitals. There was likewise no significant difference in the trend of cases of severe BA or deaths due to BA in the four hospitals, either individually or combined **(****Table 2****).**

**Table 2:** Trends in number of cases of BA and mortality in newborns with BA over 18 months in the four hospitals combined

Quarterly period	Pre-intervention (October to December 2019	Q1 (January to March 2020)	Q2 (April to June 2020)	Q3 (July to September 2020)	Q4 (October to December 2020)	Q5 (January to March 2021)	Total
Total live births	2223	2082	2311	1869	2220	1997	12702
BA cases (% of live births)*	460 (20.7)	406 (19.5)	407 (17.6)	360 (19.3)	416 (18.7)	347 (17.4)	2397 (18.9)
Severe BA (% of BA cases)†	64 (13.9)	49 (12.1)	58 (14.3)	59 (16.4)	70 (16.8)	52 (15.0)	352 (14.7)
Deaths in neonates with BA (%)	7 (1.5)	11 (2.7)	14 (3.4)	11 (3.1)	19 (4.6)	9 (2.6)	71 (2.9)

BA – birth asphyxia

*One-minute Apgar score <7.

†One-minute Apgar score ≤3.

**Figure 1:**
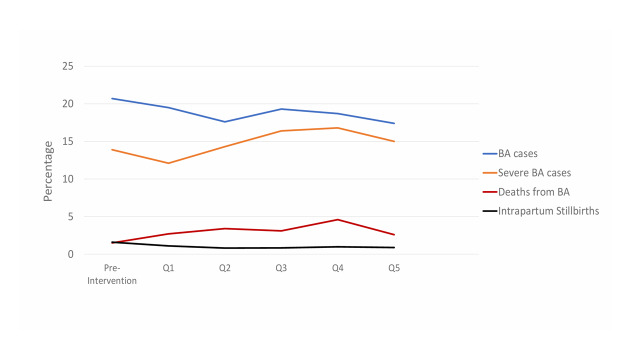
Trends in percentage of cases of BA, severe BA, deaths from BA, and intrapartum stillbirths in the four Ghana hospitals combined. BA – birth asphyxia.

The incidence of BA in the four hospitals declined from 20.7% in October to December 2019 to 17.4% by January to March 2021, but this was not statistically significant (*P* = 0.107). However, during this time period, there was a 55% decline in intrapartum stillbirths, from 1.6% to 0.89% (*P* = 0.03) (**Figure 1**). There were 48 newborns with Apgar score of 0 at one minute, who then had a transient improvement in the Apgar score to 1 or more and therefore were considered to be liveborn, but 44 (92%) of these newborns died.

**Figure 2:**
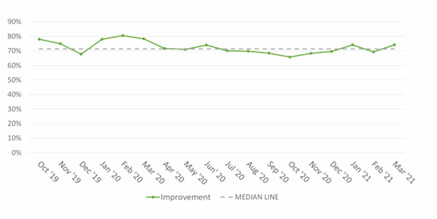
Improvement in Apgar score to 7 or more by five minutes in newborns who had one-minute Apgar scores <7 in the four Ghana hospitals over 18 months.

There was no consistent improvement in the percentage of newborns whose Apgar scores improved from <7 at one minute to ≥7 or more at five minutes over the 18-month study period (**Figure 2**).

**Figure 3:**
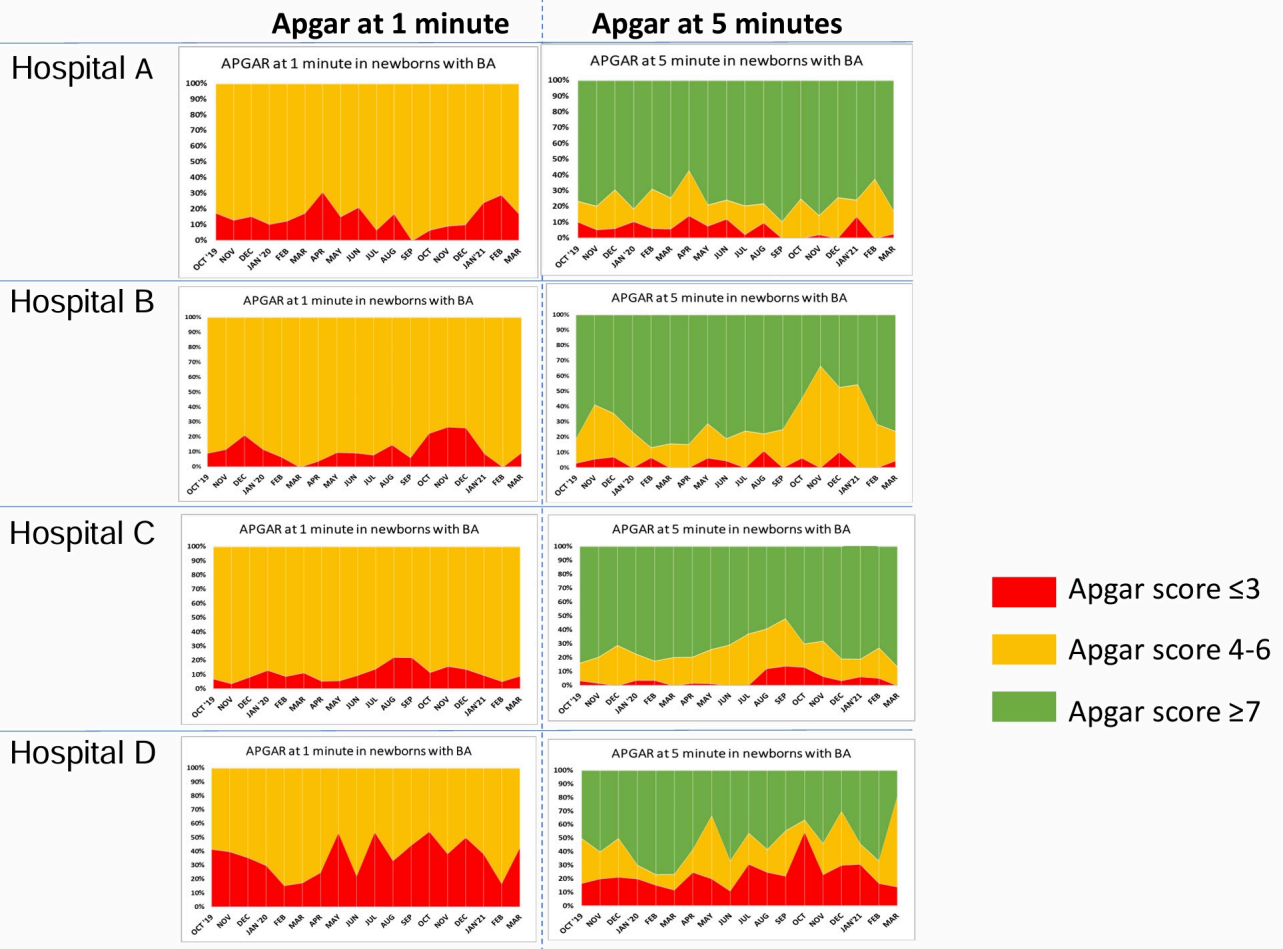
Changes in Apgar scores at 5 minutes of age in newborns who had one minute Apgar scores <7.

A detailed review of changes in Apgar score at five minutes in newborns who had Apgar scores <7 at one minute in the four hospitals shows that many newborns who started with scores of ≤6 at one minute, attained scores of ≥7, but a proportion of newborns continued to have scores of ≤3 at five minutes (**Figure 3**). Hospital A showed improvement in the 5-minute Apgar scores over time (*P* = 0.022), but hospitals B (*P* = 0.017) and D (*P* = 0.005) showed a decline over time, while hospital C showed no change. Taken together, the improvement in Apgar scores over time was not significant (*P* = 0.26). None of the four hospitals achieved a statistically significant improvement in the Apgar score to ≥7, and when combined, the overall trend was worse (*P* = 0.037).

There was a significant difference in mortality rates in newborns with Apgar scores ≤3 at one minute and those with scores of 4–6 at one minute in the four hospitals (**Table 3**). In newborns with a one-minute Apgar score ≤3, the mortality rate was significantly higher (31.5%) in those whose scores remained ≤3 than in those whose scores rose to 4–6 or more by five minutes of age (4.4%), (OR = 19.93; 95% CI = 9.4–42.1, *P* < 0.0001). Among newborns with one-minute Apgar scores of 4–6, the mortality rate in those whose five-minute Apgar score remained at ≤6 was significantly higher than in those whose five-minute Apgar score was ≥7 (OR = 5.58; 95% CI = 2.1–14.9, *P* < 0.001). Overall, the mortality rate in newborns with one-minute Apgar scores of ≤3 was significantly higher than in those with one-minute Apgar scores of 4–6 (OR = 23.48; 95% CI = 13.3–41.5, *P* < 0.001). However, the mortality rates observed here are likely underestimated, since no deaths were reported from hospital B, which transferred all its critically ill newborns to a different hospital. In 9 (0.44%) newborns with a one-minute score of 4–6, the five-minute score decreased to ≤3 at five minutes, but none died.

**Table 3:** Apgar scores at five minutes and mortality in newborns ≥34 weeks gestation with one-minute Apgar scores ≤3 or 4–6

	One-minute Apgar score ≤3 (n = 352)	One-minute Apgar score 4–6 (n = 2045)
**Five-minute Apgar score ≤3**	146 (41.5)	9 (0.44)
Mortality	46 (31.5)*	0
**Five-minute Apgar score 4–6**	195 (55.4)	307 (15.0)
Mortality	9 (4.6)*	8 (2.6)†
**Five-minute Apgar score ≥7**	11 (3.1)	1728 (84.5)
Mortality	0	8 (0.5)†
**Overall mortality**	55 (15.6)‡	16 (0.78)‡

*P < 0.001 when comparing the two indicated variables.

†*P* < 0.001 when comparing the two indicated variables.

‡*P* < 0.001 when comparing the two indicated variables.

## DISCUSSION

Since the Apgar score was introduced as a new method of evaluation of newborns soon after birth in 1953 [4], it has become a standard in hospitals in the USA and in countries around the world, including low- and middle-income countries (LMICs) [7-11].The AAP cautions against the use of the Apgar score alone to diagnose asphyxia [12], but in resource-limited countries with limited foetal monitoring or no cord blood gases, it can still provide an objective measure of the baby’s condition at birth. The WHO defines BA simply as the failure to establish breathing at birth [1], but the Apgar score provides more granular detail about breathing, heart rate, colour, tone, and reflexes in the first few minutes after birth. Although Apgar scoring begins only at one minute of age, whereas resuscitation measures like bag and mask ventilation must begin immediately, as soon as it is recognised that the infant is not breathing after birth and not responding to drying and stimulation, a low Apgar score at one minute still provides a valuable assessment of the baby’s condition at birth.

The five-minute Apgar score provides an assessment of the response to the resuscitation which may be influenced by several factors, including the gestational age, maternal medications, and, most importantly, the severity of the infant’s perinatal depression and the effectiveness of the resuscitation provided. The five-minute Apgar score has implications for mortality and morbidity in the term and near- term newborn. Large population-based studies have shown that low five-minute Apgar scores are associated with increased neonatal mortality [11,13,14], and there is epidemiological evidence that low five-minute Apgar scores are associated with adverse neurodevelopmental outcomes [15]. The Neonatal Encephalopathy and Neurologic Outcome Report defines a five-minute Apgar score of ≥7 as reassuring, a score of 4–6 as moderately abnormal, and a score of ≤3 as low in the term infant and late-preterm infant [16].

Here, we reviewed the outcomes of term and late preterm newborns born in four hospitals in Ghana, using Apgar scores of <7 at one minute as a surrogate for BA. Almost 19% of the newborns needed help to begin breathing at birth, while 2.9% had significantly more depression as indicated by the Apgar scores of ≤3. In contrast, in high income countries, it is estimated that approximately 10% of newly born infants need help to begin breathing at birth, and approximately 1% need intensive resuscitative measures to restore cardiorespiratory function [17]. In the four hospitals in Ghana, 68 to 80% of newborns with Apgar scores less than 7 at one minute attained scores of 7 or more at five minutes in the pre-intervention phase between October and December 2019, but there was no consistent improvement in the post-intervention phase. Deeper examination revealed that although many of the infants with Apgar scores <7 improved, most of the improvements were among newborns who had scores of 4–6, with 85% attaining scores of ≥7 at five minutes, indicating that HBB measures were effective in these infants. However, among babies with Apgar scores ≤3, the score remained at ≤3 at five minutes in 41.5% and a third of these infants died. In the significant proportion of these infants (55%) who attained scores of at least 4 at five minutes, the mortality rate was < 5%, showing that HBB is effective in at least half of these infants.

Recent reviews of HBB programmes in several countries have shown that implementation of HBB was associated with decreased perinatal mortality in most, but not all studies [18,19]. In southern India, HBB was associated with a decrease in stillbirths, but there was no difference in neonatal mortality [20]. In a study of 70 704 births in facilities in India and in Kenya, there was no significant impact on perinatal or neonatal mortality in normal weight newborns despite rigorous HBB training [21]. In contrast, a recent study in Nepal showed significant improvement in both stillbirth rates and early neonatal deaths with HBB training [22]. 

We noted a significant decrease in stillbirth rates over the 18 months. The conversion from an apparent stillbirth to a live birth is the detection of any sign of life, usually a heartbeat, even if transient. This may be easier to achieve than significantly decrease neonatal mortality. The 92% mortality in live-born infants with Apgar scores of 0 at 1 minute indicates that an attempt is being made to resuscitate apparently stillborn infants, but many of them still perish, and the survivors are likely to be at high risk for poor neurological outcomes. We were unable to show a decrease in mortality in live-born infants over the same time period. The high rates of severe BA in this study may be due to several factors that need to be elucidated, including delayed care seeking during the COVID pandemic.

Simple basic resuscitation was introduced by the WHO in 1997, followed by other programs including US Agency for International Development Basic Institutionalization of Child Survival based on the premise that only 2% of babies required the additional components of advanced resuscitation, namely endotracheal intubation, cardiac massage, and medications [23]. The AAP HBB was disseminated globally in 2010, focussing on bag and mask ventilation in the Golden Minute [2]. However, the high incidence of severe BA and mortality in infants who had Apgar scores of ≤3 at five minutes of age would suggest that perhaps more intensive resuscitation including intubation, chest compressions, and epinephrine administration in health care facilities, as taught in the Neonatal Resuscitation Program, could have led to an improved five-minute score and potentially a decrease in neonatal mortality. A laryngeal mask airway provides more efficient ventilation than face mask ventilation, but the trials so far in LMICs have not shown a significant difference in outcome in babies resuscitated with a laryngeal mask airway *vs.* standard face mask ventilation [24]. Moreover, a population-based study of term infants in Norway, a high-resource country, showed that if both the one- and five-minute Apgar scores were ≤3, the risk for neonatal death increased 642 fold compared with scores of ≥7, indicating that advanced resuscitation is not always the final answer to babies born with low Apgar scores [25].In addition, infants requiring more intensive or prolonged resuscitation are at risk for immediate and long term complications [16] A recent review indicated that mortality and morbidity in intrapartum-related neonatal encephalopathy has been static in the last 10 years in LMICs [26].

In view of our findings, we believe the Apgar score can be the basis for both evaluating existing interventions and planning more effective ones to reduce neonatal mortality due to BA. A high proportion of infants being born with Apgar scores of ≤3 indicates that better antenatal and intrapartum care, in addition to conventional resuscitation at birth, is required to reduce neonatal mortality and morbidity. Counselling mothers to reach the facility at the appropriate time in labour, establishing more efficient transport systems, and improving skills in obstetric management (notably addressing the second stage of labour, including foetal heart rate monitoring) with early intervention for non-reassuring foetal heart states may lead to babies being born in better condition. Combining HBB training programmes with relevant modules of Helping Mothers Survive, focussing on labour and delivery could potentially help reduce stillbirth and early neonatal mortality rates [27]. The use of a novel foetal heart rate monitor, coupled with HBB training at lower health facilities in Tanzania, resulted in a decrease in fresh stillbirths and early neonatal deaths [28].

The limitations of this study include the short period of assessment of only 18 months which overlapped with the COVID-19 pandemic, which undoubtedly impacted the staffing in labour and delivery rooms in the health facilities. Most population-based research studies report on the impact of resuscitation by demonstrating the decrease in stillbirths or neonatal deaths per 1000 births over a longer time period [22]. There was also significant inter-hospital variation in the incidence of BA and BA-related mortality in the four hospitals in Ghana; however, without detailed data about staffing and patient population characteristics, we cannot speculate on the causes for this. In a QI exercise in smaller hospitals, where the number of deaths each month is small, major fluctuations may be noted in run charts. We attempted to smooth that by combining data from all four hospitals.

While we could not show improvement in survival of newborns with HBB, one strength of this study lies in the selection of late preterm and term newborns as the population of interest. This contrasts with most other HBB studies, where all newborns were included and where mortality due to prematurity cannot be separated from deaths due to BA. The neonatal mortality rates observed here were comparable to post-HBB neonatal mortality in other studies [18-20], but were not as low as the one-day neonatal mortality reported post-HBB in Nepal [22]. One-day neonatal mortality may not reflect the full scale of mortality associated with BA. However, our study was on infants ≥34 weeks gestational age, so it may underestimate neonatal mortality compared to other research. Furthermore, one hospital reported no deaths, since critically ill newborns were transferred to another hospital, suggesting that the mortality rate in our study was likely higher. Nevertheless, documenting the details of Apgar scores provided critical information on which babies survived and where improvements need to be made to ensure survival in the future.

## CONCLUSIONS

Despite the well-known limitations of the Apgar score, properly documented scores can be beneficial, especially in LMICs. Studying the changes in the Apgar scores between one minute and five minutes of age can be useful in documenting the improvement with resuscitation, especially when pulse oximeters, cardiac monitors, and cord blood gases are not available. A high proportion of infants with an Apgar score of ≤3 at one minute suggests that improving the skills of providers performing resuscitations and including more advanced resuscitation is necessary in health care facilities. More importantly, it highlights the need for policies to provide an integrated approach for management of maternal and newborn care starting with antenatal care, improved transport systems, and appropriate intrapartum care, followed by adequate resuscitation and post-resuscitation care [29].
